# Sarcopenia in open heart surgery patients: A cohort study

**DOI:** 10.1016/j.heliyon.2020.e05759

**Published:** 2020-12-17

**Authors:** Kornanong Yuenyongchaiwat, Chitima Kulchanarat, Opas Satdhabudha

**Affiliations:** a Physiotherapy Department, Faculty of Allied Health Sciences, Thammasat University, Thailand; b Thammasat University Research Unit in Physical Therapy in Respiratory and Cardiovascular Systems, Thailand; c Division of Cardiovascular Thoracic Surgery, Department of Surgery, Thammasat University Hospital, Thailand

**Keywords:** Cardiology, Musculoskeletal system, Surgery, Internal medicine, Clinical research, Sarcopenia, Open heart surgery, Prevalence

## Abstract

**Background:**

Sarcopenia is a condition characterized by loss of muscle mass, muscle strength, or physical performance. It has been reported that cardiac surgery causes systemic inflammatory response, which leads to sarcopenia. In addition, open-heart surgery (OHS) has been associated with length of hospital stay, prolonged mechanical ventilation, and postoperative pulmonary complications. However, very few studies have explored the association of sarcopenia with OHS. Thus, this study explores the prevalence of sarcopenia in OHS patients as well as their relationship.

**Methods:**

This cohort study included 160 patients; it was designed to assess sarcopenia during preoperative OHS and before patient discharge from the hospital. Sarcopenia was defined according to Asian Working Group for Sarcopenia (AWGS) criteria as low muscle mass plus low muscle strength and/or slow gait speed. Participants were requested to perform exercises to test their handgrip strength, gait speed, and bioelectrical impedance. In addition, their medical history (e.g., duration of hospitalization and mechanical ventilation) was recorded.

**Results:**

The prevalence of sarcopenia during preoperative OHS was 26.9%, with affected men comprising 11.9% and affected women comprising 15% of the total sample. Participants with sarcopenia had a significantly lower body mass index (BMI) than those without. Further, patients who had longer stays in the hospital and prolonged mechanical ventilation time showed significantly higher rates of developing sarcopenia. During postoperative OHS, the incidence of sarcopenia rose by 20.92%, increasing the total prevalence of sarcopenia to 46.41%. Moreover, advanced age emerged as one of the most significant risk factors of sarcopenia. Participants in the age group >55 years had an increased risk of sarcopenia (odds ratio [OR]: 3.90). It was also found that patients with a low BMI (<23 kg∗m^−2^) and a history of diabetes mellitus (DM) had an increased risk of sarcopenia (ORs: 2.11 and 1.47, respectively). Moreover, longer hospital stays and mechanical ventilation times were important risk factors (ORs: 1.58 and 2.07, respectively).

**Conclusion:**

The prevalence of sarcopenia was observed to be high during postoperative OHS. Participants with sarcopenia who underwent OHS had a history of DM, longer length of hospital stays, and prolonged mechanical ventilation times, compared with patients without sarcopenia.

**Clinical trial registration number:**

TCTR20190509003.

## 1 Introduction

Sarcopenia is a disease characterized by loss of muscle mass, muscle strength, or physical performance [1, 2]. It is a major health problem around the world and impacts people's daily activities and quality of life [1, 2, 3]. Sarcopenia can be divided into two categories: primary and secondary sarcopenia. Primary sarcopenia is associated with age and does not have any other causes; whereas secondary sarcopenia is associated with cardiovascular disease (CVD), acute coronary syndrome, and cardiac surgery [1, 2, 4]. One of the earliest studies on muscle wasting in patients with cardiac disease, by Fulster et al. in 2013, found that muscle wasting (defined as a muscle mass values less than -2 SD below the mean of a healthy young reference group of adults aged 18–40 years) was noticed in 19.5% in chronic heart failure [5]. Further, it has been found that the prevalence of sarcopenia in patients with CVD was 29.7%, comprising 48.7% of women and 19.6% of men [2]. Moreover, Kamiya et al. reported that sarcopenia occurs in 17.8% of patients with acute coronary syndrome and 31.8% of post-cardiac surgery patients [2].

Cardiac surgery causes a systemic inflammatory response associated with sarcopenia [2]. This inflammatory response releases cytokines, which play an important role in muscle breakdown leading to loss of muscle mass and sarcopenia [2, 6]. Okamura et al. found the prevalence of sarcopenia to be 20.0% in patients during preoperative valve surgery [7]. Another study showed the prevalence of sarcopenia in patients with post-cardiac surgery was 31.8% [2]. In addition, the prevalence of sarcopenia in patients during the first year after cardiac surgery was 27.7% [8]. Previous studies have reported the prevalence of sarcopenia post-cardiac surgery; however, only a few have studied the association of sarcopenia with open-heart surgery (OHS), which is influenced by length of hospital stay, prolonged mechanical ventilation, and complications after cardiac surgery [2, 4]. Moreover, no studies have examined the incidence and prevalence of sarcopenia in Thailand. Therefore, the purpose of this study was to determine the prevalence of sarcopenia among patients who have had OHS, as well as the association of sarcopenia with OHS.

## 2 Materials & methods

This cohort study was designed with 160 preoperative OHS patients as its participants. The study protocol was approved by the Ethics Committee of Thammasat University (approval reference no. 057/2562). All participants were required to complete an informed consent form prior to the study.

All recruited individuals were between 35 and 80 years old, were preoperative OHS patients, and were scheduled to undergo median sternotomies. Participants with unstable angina or uncontrollable arrhythmia, a resting heart rate above 120 bpm, resting systolic blood pressure above 180 mmHg, and/or diastolic blood pressure above 100 mmHg within 48 h prior to the study were excluded. In addition, patients who had undergone other surgeries, such as thoracotomies (e.g., lobectomies and pneumonectomies), and those with musculoskeletal disorders that resulted in an inability to walk (e.g., osteoarthritis, inflammatory arthritis, edema, and neurological disease, such as paralysis of the lower limbs or hemiparesis due to stroke or traumatic brain injury), or had undergone limb amputation were also excluded.

According to the 2018 data from the heart operation unit of a hospital, the number of patients who underwent OHS was 241 [9]. The sample size was calculated at 150 patients, but in order to preempt any dropouts, 160 patients were recruited.

The participants’ medical history (e.g., New York Heart Association [NYHA] classification and history of underlying disease), duration of hospitalization after postoperative heart surgery, and duration of being on a mechanical ventilator were recorded. The length of hospitalization after postoperative heart surgery was determined from the date of operation to the date of discharge from the hospital, which was within 48 h from the former. The definition of sarcopenia, which was proposed by the Asian Working Group for Sarcopenia (AWGS), is the loss of muscle mass plus low physical performance and/or muscle strength [10]. For the present study, sarcopenia was diagnosed when individuals met the following criteria: low muscle mass plus poor physical performance (i.e., slow gait speed) and/or decreased muscle strength (poor handgrip strength). Therefore, these measures were assessed before and after OHS.

Handgrip strength was measured using a handgrip dynamometer. It should be noted that the handgrip dynamometer has test-retest reproducibility (r > 0.80) and intra-rater reliability (r = 0.98) [11]. Participants were required to stand with their elbow extended and hold the handgrip dynamometer for three seconds. The maximum score was recorded. According to the recommendation of AWGS, low handgrip strength is determined as <26 kg for males and <18 kg for females [10].

The 6-meter walk test was used to measure gait speed. The test was repeated three times, and the best value was recorded. Slow gait speed was determined as <0.8 m per second for both men and women [10].

Finally, muscle mass was measured through bioelectrical impedance analysis (BIA). This method was chosen as it utilizes an inexpensive device and portable equipment, and it has been accepted under the guidelines of the European Working Group on Sarcopenia in Older People (EWGSOP) and AWGS [10, 12, 13]. The muscle mass was calculated using the following BIA equation of Janssen et al [14] which is

$$\text{Skeletal muscle mass }\left(\text{kg}\right)=\left[\left({\text{Ht}}^{2}/\text{BIA resistance }\times \;0.401\right)+\left(\text{gender}\times 3.825\right)+\left(\text{age}\times -0.071\right)\right]+5.102$$

where Ht is height in centimeters; BIA resistance is resistance in ohms:

For gender, men = 1 and women = 0 and age is years.

Further, the BIA equation was strongly associated with magnetic resonance imaging (MRI)-measured skeletal mass [14].

In addition, skeletal muscle ratio (%) = [skeletal muscle mass (kg)/body weight (kg)] X 100. Muscle mass index was calculated by percentage of skeletal muscle multiplied by their body weight and then divided by their height in m^2^.

Participants were not allowed to exercise 30 min before the test. Furthermore, they were asked to abstain from food, beverage, and alcohol consumption 2 h prior to the test. They were also requested to go to the restroom to provide a urine sample. During the test, the participants were required to stand on their bare feet, and were tasked with grasping the electrode with both hands. Body weight and skeletal muscle percentage readings were evaluated by using BIA (body composition monitor HBF-375). Therefore, low muscle mass was determined as <7.0 kg/m^2^ for men and <5.7 kg/m^2^ for women [10].

All data were analyzed using SPSS Version 23.0 software. Descriptive statistics was used to analyze the features of the sample population and to report them in mean and standard deviation, frequency, and percentage. The Kolmogorov-Smirnov statistic was analyzed for normality of distribution. The t-test and chi-squared test were conducted where appropriate, with the level of significance set at less than 0.05. In addition, logistic regression analysis was performed to determine the predictive factors for sarcopenia after OHS (length of hospital stay, prolonged mechanical ventilation, and complications).

## 3 Results

**Figure 1:**
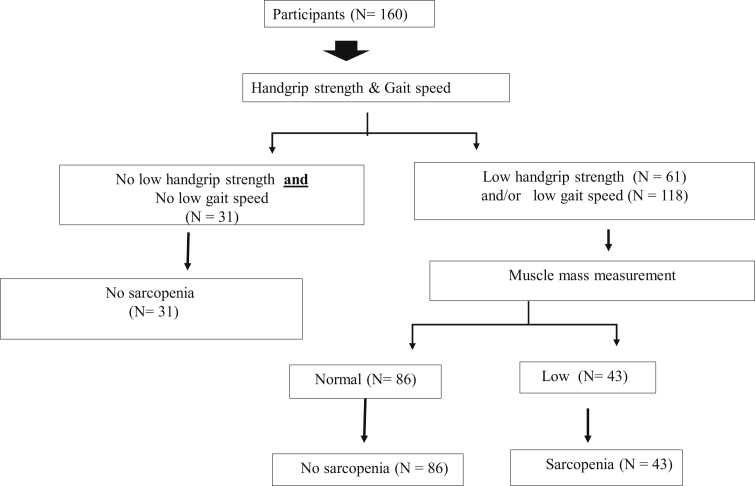
Sarcopenia category by diagnostic algorithms of Asian Working Group for Sarcopenia at preoperative open heart surgery.

Figure 1 displays sarcopenia categories using diagnostic algorithms of AWGS during preoperative OHS. Among the 160 participants (96 men and 64 women), sarcopenia was present in 43 patients (26.88 %). Among those with sarcopenia during preoperative OHS, 11 (6.88%) were deemed to have sarcopenia because of poor grip strength (n = 5, 3.13%) or low gait speed (n = 6, 3.75%), whereas 32 (20.00%) had decreased muscle strength and slow gait speed concomitantly.

**Figure 2:**
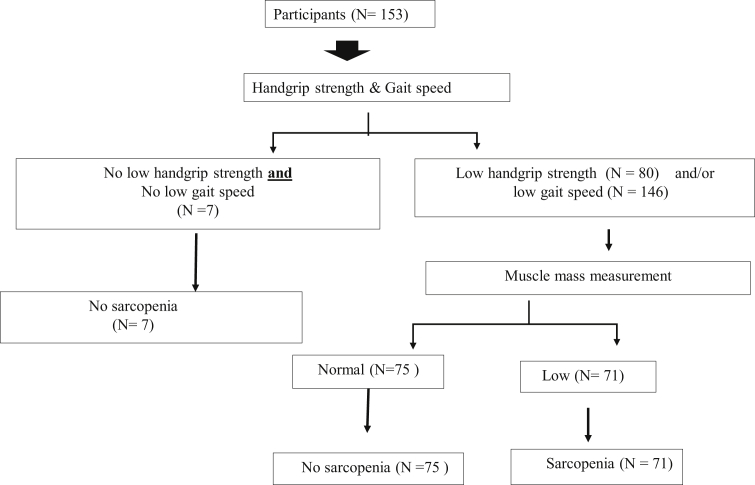
Sarcopenia category by diagnostic algorithms of Asian Working Group for Sarcopenia at post-operative open heart surgery.

**Figure 3:**
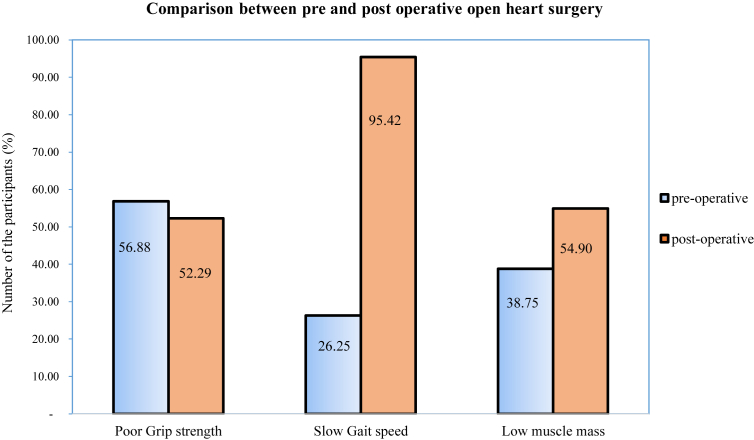
Comparison of grip strength, gait speed and muscle mass between pre and post operative open heart surgery.

From 160 patients during preoperative OHS, 7 patients were not recruited post-operation, 6 patients died after postoperative heart surgery (3 patients had sarcopenia and 3 did not), and 1 patient with sarcopenia was discharged prior to data collection. Ultimately, 153 participants were enrolled after postoperative OHS. After postoperative OHS, it was found that the incidence of sarcopenia increased by 20.92% (n = 32). Overall, the prevalence of sarcopenia in patients during postoperative OHS was 46.41% (n = 71). Among them, 9 (5.88%) were sarcopenic because of low gait speed, whereas 62 (40.52%) had the concomitant presence of decreased muscle strength and slow gait speed. Only 7 participants showed no low grip strength and low gait speed (Figure 2). Further, there was an increase in the number of OHS patients who showed slow gait speed and reduced muscle mass, compared with the preoperative period (Figure 3).

The average age of the study population was 61.1 ± 11.5 years. Participants with sarcopenia were significantly older compared to those without sarcopenia. Among the sarcopenic group, 32 patients (20%) were classified as NYHA class II; NYHA class IV was not reported in the medical records. Regarding the comorbidities in the sarcopenic group, participants with sarcopenia were significantly affected by only a history of DM (χ^2^ = 79.558, *p* < 0.001).

Time spent while on a mechanical ventilator also significantly differed among patients who developed sarcopenia and those who did not (872.2 ± 287.9 min and 723.7 ± 293.5 min, respectively; *p* < 0.001). Similarly, length of hospital stay was significantly longer among patients who developed sarcopenia than those who did not (9.3 ± 3.8 days and 7.2 ± 2.0 days, respectively; *p* < 0.001) (Table 1).

**Table 1:** Characteristics data in pre-operative open heart surgery patients (N = 160).

	Total (%) N = 160	Pre-operative		*p* value^a^
		Sarcopenia (%)	Non sarcopenia (%)	
		N = 43	N = 117	
Gender				0.19
Male	96 (60.0)	19 (11.9)	77 (48.1)	
Female	64 (40.0)	24 (15.0)	40 (25.0)	
Alcohol consumption	71 (44.4)	15 (9.4)	56 (35.0)	0.14
History of smoking	66 (41.3)	16 (10.0)	50 (31.3)	0.52
Classification of NYHA class				0.58
I	21 (13.1)	4 (2.5)	17 (10.6)	
II	113 (70.6)	32 (20.0)	81 (50.6)	
III	26 (16.3)	7 (4.4)	19 (11.9)	
Surgical procedures				0.22
CABG	89 (55.6)	21 (13.1)	68 (42.5)	
Valve surgery	61 (38.1)	18 (11.3)	43 (26.9)	
CABG plus valve surgery	10 (6.3)	4 (2.5)	6 (3.8)	
Comorbidities				
Hypertension	113 (70.6)	34 (21.3)	79 (49.4)	0.15
Diabetes mellitus	68 (42.5)	43 (26.9)	25 (15.6)	<0.001
Dyslipidemia	65 (40.6)	20 (12.5)	45 (28.1)	0.35
Chronic kidney disease	24 (15.0)	9 (5.6)	15 (9.4)	0.20
	Mean ± SD	Mean ± SD	Mean ± SD	*p* value^b^
Age (years)	61.1 ± 11.5	66.4 ± 10.7	59.1 ± 11.2	0.001
BMI (kg/m^2^)	23.8 ± 4.3	20.8 ± 3.0	24.8 ± 4.2	0.04
Muscle mass index (kg/m^2^)	6.5 ± 1.7	5.1 ± 1.0	7.0 ± 1.7	<0.001
Gait speed (m/s)	0.7 ± 0.2	0.6 ± 0.2	0.7 ± 0.2	0.005
Handgrip strength (kg)	24.7 ± 8.5	17.5 ± 5.5	27.3 ± 7.9	<0.001
LVEF (%)	52.3 ± 14.0	53.3 ± 15.1	51.9 ± 13.7	0.46
Duration of surgery (min)	342.8 ± 131.4	324.9 ± 138.3	349.4 ± 128.8	0.29
Duration of mechanical ventilator (min)	852.3 ± 262.6	872.2 ± 287.9	723.7 ± 293.5	<0.001
Length of hospital stay (days)	7.8 ± 2.8	9.3 ± 3.8	7.2 ± 2.0	<0.001
CPB time (min)	110.2 ± 54.9	112.28 ± 56.9	104.53 ± 49.4	0.431

NYHA; New York Heart Association, CABG; coronary artery bypass grafting, BMI; body mass index, LVEF; left ventricular ejection fraction, CPB; cardiopulmonary bypass.

Note: length of hospital stay was calculated from 153 participants (sarcopenia = 40 and non-sarcopenia = 113).

a analyzed by chi-square test.

b analyzed by t-test.

Logistic regression analysis was performed on risk factors associated with sarcopenia (i.e., age, DM, and BMI), length of hospital stay, and the duration of mechanical ventilation. The results showed that mechanical ventilation for more than 24 h and a hospital stay longer than 5 days were significantly associated with an increased risk of sarcopenia (odds ratio [OR] = 2.07, *p* = 0.04 and OR = 1.57, *p* = 0.01, respectively). In addition, participants over 55 years of age and older had a higher risk of sarcopenia compared with those below 55 years old. BMI was significantly lower than 23 kg/m^2^ among participants in the sarcopenic group (OR = 2.11, *p* = 0.04). Furthermore, participants with DM were significantly associated with an increased risk of sarcopenia (OR = 1.47, *p* = 0.02); see Table 2.

**Table 2:** Risk of factors sarcopenia in open heart surgery patients.

Risk factors	Odds Ratio	95% CI for OR	p-value
	Reference age group ≤55 years		
Age >55 years	3.90	2.01–7.89	0.002
	Reference BMI ≥23 kg/m^2^		
BMI <23 kg/m^2^	2.11	1.00–4.46	0.04
Comorbidities	Reference no history of DM		
DM	1.47	0.85–2.32	0.02
	Reference LOS ≤5 days		
LOS >5 days	1.57	0.81–3.06	0.01
	Reference ventilation ≤24 h		
Mechanical ventilation >24 h	2.07	0.97–4.40	0.04

BMI; body mass index, DM; diabetes mellitus, LOS; length of stay in the hospital.

## 4 Discussion

This study aimed to determine the prevalence of sarcopenia in OHS patients as well as the association between OHS and sarcopenia. The risk factors for sarcopenia were age, low BMI, DM, prolonged mechanical ventilation, and length of hospital stay. To our knowledge, this is one of the first studies to assess sarcopenia during both preoperative and postoperative OHS. A high prevalence rate of sarcopenia was observed in the present study, compared with previous studies. This may be attributed to the different methods of assessing sarcopenia (e.g., computed tomography scans, dual energy x-ray absorptiometry) as well as the different diagnostic criteria (e.g., EWGSOP, AWGS).

The present study found that the older age group was at a higher risk of sarcopenia during preoperative OHS compared to the younger age group; these findings are consistent with epidemiological studies that found an association between age and loss of skeletal muscle and sarcopenia [15, 16]. In addition, sarcopenia in preoperative heart surgery patients has been reportedly associated with age, longer hospital stay, low physical performance, and functional limitations in relation to sarcopenia.

Our results revealed that patients with low BMI often exhibited sarcopenia. Malnutrition, such as a decrease in albumin levels, is generally reported in patients after OHS [17, 18, 19, 20, 21]. Meanwhile, the systemic inflammatory response to cardiac surgery may account for the low BMI of sarcopenic patients. Moreover, this process can also lead to organ dysfunction syndrome, and death after OHS [22].

In our study, patients with DM were found to be associated with an increased risk of sarcopenia. This may be due to the fact that patients with DM have a higher risk of low muscle mass and decreased muscle strength. In addition, high blood glucose levels accelerate the reduction of muscle mass, muscle strength, and physical performance—all of which contribute to sarcopenia [23]. Changes in inflammatory mediators (e.g., inflammatory cytokines and adipokines) are linked to metabolic hormone reactions, including insulin and glucagon [24]. Additionally, inflammatory markers, such as IL-6 are related to diabetes risk, metabolic syndrome, and an increased loss of muscle mass and muscle strength [25]. Therefore, further studies must find evidence to support a mechanism linking physiological interrelated factors to sarcopenia in patients undergoing OHS.

Our study suggests that the duration of mechanical ventilation is associated with sarcopenia. Several factors related to altered muscle strength and prolonged mechanical ventilation contribute to immobility in bed, sepsis, systemic inflammatory response syndrome—which has an adverse impact on functional status—prolonged intubation, and length of hospital stay [26, 27, 28]. In addition, other factors, such as respiratory complications, may increase the duration of mechanical ventilation time [29]. Therefore, mechanical ventilation time is associated with a longer hospital stay and leads to an increased incidence of sarcopenia in OHS patients. It may be provoked by vigorous systematic inflammatory response due to the release of hormones and cytokines [30]. Proinflammatory cytokines cause an increase in protein degradation and a decrease in protein synthesis; this decline in protein synthesis leads to loss of muscle mass and muscle strength [30]. Thus, it may be said that muscle proteolysis contributes to decreased physical performance and loss of muscle mass after CABG [31] and resulting in prolong mechanical ventilation or long duration of hospital admission.

## 5 Limitations of this study

This study had some limitations that might have affected its results. First, data regarding NYHA class IV were not reported. This may have affected data regarding the prevalence of sarcopenia, length of hospital stay, and prolonged mechanical ventilation within the study. In addition, the levels of proinflammatory cytokines, such as IL-6 and TNF-α, were not examined. Finally, even though improvements in exercise or physical activity may increase muscle mass and reduce the risk for sarcopenia [32], the present study did not account for nor examine this factor. Therefore, future studies should explore the mechanism of sarcopenia in OHS patients and the benefits, if any, of exercise or physical activity.

## 6 Conclusion

Sarcopenia is a highly prevalent and incidental syndrome in patients after OHS. Advanced age, low BMI, history of DM, long duration of hospital stay, and prolonged mechanical ventilation were significantly more common in OHS patients with sarcopenia than in those without.

## Declarations

### Author contribution statement

K. Yuenyongchaiwat: Conceived and designed the experiments; Performed the experiments; Analyzed and interpreted the data; Contributed reagents, materials, analysis tools or data; Wrote the paper.

O. Satdhabudha: Conceived and designed the experiments.

C. Kulchanarat: Performed the experiments; Analyzed and interpreted the data; Contributed reagents, materials, analysis tools or data; Wrote the paper.

### Funding statement

This research did not receive any specific grant from funding agencies in the public, commercial, or not-for-profit sectors.

### Declaration of interests statement

The authors declare no conflict of interest.

### Additional information

The clinical trial described in this paper was registered at Thai Clinical Trials Registry under the registration number TCTR20190509003.
